# Prevalence, predictors, dynamic bone change, and treatment efficacy of osteoporosis among chronic obstructive pulmonary disease patients: a prospective cohort study

**DOI:** 10.3389/fmed.2023.1214277

**Published:** 2023-08-08

**Authors:** Punchalee Kaenmuang, Warangkana Keeratichananont, Sarayut Lucien Geater, Nicha Chantamanee, Piyaporn Srikaew

**Affiliations:** ^1^Respiratory and Respiratory Critical Care Medicine Unit, Division of Internal Medicine, Faculty of Medicine, Prince of Songkla University, Hat Yai, Songkhla, Thailand; ^2^Division of Internal Medicine, Faculty of Medicine, Prince of Songkla University, Hat Yai, Songkhla, Thailand

**Keywords:** chronic obstructive pulmonary disease (COPD), osteoporosis, bone mineral density (BMD), bisphosphonate, alendronate

## Abstract

**Background:**

Osteoporosis is a silent chronic obstructive pulmonary disease (COPD) comorbidity that is often under-detected. We aimed to study the prevalence and potential predictors of osteoporosis in COPD. Dynamic changes in bone mass density (BMD) and treatment efficacy of bisphosphonate were also assessed.

**Methods:**

This prospective cohort study included COPD patients between January 2017 and January 2019. Demographics data, spirometric parameters, and C-reactive protein (CRP) were collected. Bone mineral density (BMD) at the lumbar spine (L2-4) and both femoral necks were measured after enrollment and the 12-month follow-up. Participants were categorized into three groups per the baseline BMD T-score: normal (≥ − 1.0), osteopenia (between −1.0 and − 2.5), and osteoporosis (≤ − 2.5). In the osteoporosis group, alendronate 70 mg/week with vitamin D and calcium was prescribed.

**Results:**

In total, 108 COPD patients were enrolled. The prevalence of osteoporosis and osteopenia were 31.5 and 32.4%, respectively. Advanced age, lower body mass index (BMI), history of exacerbation in the previous year, and high CRP levels were significant predictors of osteoporosis. After 12 months, 35.3% in the osteoporosis group reported new vertebral and femoral fractures, compared to none in the non-osteoporosis group (*p* < 0.001). In the normal BMD and osteopenia groups showed a further decline in BMD after 12-month. Conversely, the osteoporosis group showed a statistically significant improvement in BMD after anti-resorptive treatment (*p* < 0.001).

**Conclusion:**

The prevalence of osteoporosis was high in Thai COPD patients. Advanced age, lower BMI, history of exacerbation, and high CRP levels were potential predictors. A rapid decline in BMD was observed in COPD patients without treatment.

## 1. Introduction

Chronic obstructive pulmonary disease (COPD) is common and causes progressive and persistent respiratory symptoms. In 2019, COPD was the third leading cause of death worldwide and will continue rising (1). COPD patients also have concomitant chronic diseases due to systemic involvement, including cardiovascular disease, skeletal muscle wasting, lung cancer, osteoporosis, metabolic syndrome, and anxiety/depression (2). Comorbidities often affect the progression, morbidity, and mortality of patients with COPD.

Osteoporosis is a silent COPD comorbidity that is closely related and often under-detected in the clinic; it leads to poor health status and mortality. Osteoporosis is characterized by a decrease in bone mass and microarchitectural deterioration of the bone tissue, leading to bone fragility and fracture (3). However, the pathophysiology of COPD-associated osteoporosis is still not well understood and requires further study. This hypothesis was based on low bone mineral density (BMD), abnormal bone changes, impaired bone quality, and low bone turnover due to systemic inflammation (4). Nowadays, there are much increasingly diverse data on the relationship between osteoporotic risk factors and COPD; including smoking, systemic inflammation, long-term corticosteroids used, decreased physical activity, smoking, and malnutrition (5). The molecular pathways of osteoporosis in COPD patients comprise of the interaction between risk factors and molecular pathways such as inflammatory cytokines, irisin, myostatin, osteoclast differentiation, osteoblast activity, etc. (6). These purposed pathways describe the mechanisms of bone loss and muscle loss in COPD patients. Meanwhile, osteoporosis can lead to fractures that have an enormous impact; vertebral fractures may reduce pulmonary function, and rib fractures can cause hypoventilation and interfere with expectorant secretion (7). In previously published studies, the prevalence of osteoporosis in patients was reported to be approximately 23–50% (8–12). A current meta-analysis reported that the pooled global prevalence of osteoporosis COPD was 38% and that COPD increased the likelihood of osteoporosis [odds ratio (OR) = 2.83] (8). Common risk factors for osteoporosis in patients were low body mass index (BMI) and muscle mass. Old age, female sex, severe airflow limitation, frequent exacerbations, advance COPD categories, and C-reactive protein (CRP) levels were also mentioned as potential risk factors (8–14).

The current recommendations for the treatment of osteoporosis (15–17) involve non-pharmacological and pharmacological management. Bisphosphonates, an antiresorptive therapy, are the most widely used treatment for osteoporosis. They have a clear benefit in postmenopausal and glucocorticoid-induced osteoporosis. However, there is limited evidence regarding patients with osteoporotic COPD. A previous randomized controlled trial (RCT) of airway disease demonstrated a significant improvement in lumbar spine BMD through daily intake of 10 mg alendronate (18).

Currently, only one study has reported the prevalence of osteoporosis in Thai COPD patients, (11) and there is limited evidence of dynamic BMD changes in COPD patients. In addition, the role of bisphosphonates in patients with osteoporotic COPD is not well established. This study aimed to investigate the prevalence of osteoporosis in Asian (Thai) COPD patients, define potential predictors of osteoporosis, evaluate dynamic bone changes, and explore the efficacy of bisphosphonate treatment in COPD patients.

## 2. Materials and methods

### 2.1. Study populations and design

This prospective cohort study was conducted at a single tertiary hospital, which is a major referral center for 14 provinces in southern Thailand, from January 2017 to January 2019. Patients were eligible for this study if they met the following criteria: age ≥ 40 years, stable COPD diagnosed according to the Global Initiative for Chronic Obstructive Lung Disease (GOLD) guideline 2017, and followed up at the outpatient department (19). Patients were excluded if they had acute exacerbation within 2 months, had comorbidities that could affect bone metabolism e.g., chronic kidney disease (CKD) ≥ stage 3, malignancy, chronic granulomatous disease, hyperparathyroidism, hepatic impairment or chronic liver disease, endocrinal disorders (type 1 diabetes mellitus, Addison’s disease, Cushing’s syndrome, and Graves’ disease), bone metabolism disorders (Paget’s disease and osteogenesis imperfect), mastocytosis, severe malabsorption, or received medication related to bone metabolism, including bisphosphonate, systemic glucocorticoids, and hormonal replacement.

Demographic data, including age, sex, BMI, underlying disease, smoking history, severity of COPD, exacerbation rates in the previous year, and inhaled corticosteroid use, were collected. C-reactive protein (CRP) levels were obtained and measured at the central laboratory of Songklanagarind Hospital, using turbidimetric/immunoturbidimetric methods (SENTINEL CH. S.p.A., Milano, Italy).

BMD measurements were performed in three areas: the lumbar spine (L2-4) and both femoral necks, using dual energy X-ray absorptiometry (DXA scan; PRODIGY Pro, GE healthcare/U.S.) after enrolment and a 12-month follow-up by a well-trained technician. Osteoporotic fracture events were also recorded during the follow-up period. All participants were categorized into three groups as follows: normal (≥ − 1.0), osteopenia (between − 1.0 and − 2.5), and osteoporosis (≤ − 2.5) according to the overall baseline BMD based on the World Health Organization (WHO) recommendations (20). COPD patients with osteoporosis were prescribed alendronate 70 mg/week, calcium 2,500 mg/day, and vitamin D 20,000 IU/week.

This study was approved by the Office of Human Research Ethics Committee at the Faculty of Medicine, Prince of Songkla University, Thailand (REC.57–225–14-1). All patients provided written informed consent prior to enrollment.

### 2.2. Outcomes

The primary outcome was the prevalence of osteoporosis in Thai COPD patients. Secondary outcomes were the potential predictors of osteoporosis and natural dynamic changes in BMD (the differences in baseline and 12-month follow-up BMD in the non-osteoporotic group) in patients with COPD. The treatment efficacy of bisphosphonate was also assessed by the percentage difference between the baseline and 12-month follow-up BMD in the osteoporotic group.

### 2.3. Statistical analyses

The sample size was calculated using an infinite population proportion from the application called n4Studies (21) according to previously published studies on the prevalence of osteoporosis in patients with COPD (11). A total sample size of 103 patients, which included an additional 20% with missing data, was analyzed in this study.

Continuous demographic data are reported as mean ± standard deviation (SD) or median with interquartile range (IQR). Discrete parameters are presented as counts and percentages. Inferential statistics were used to compare the patient characteristics and outcomes. The chi-square test was used to compare differences in categorical variables, whereas Student’s t-test (Kruskal-Wallis equality-of-populations rank test) was used for continuous variables.

Seemingly unrelated regression (SUR) analysis was used to minimize confounding factors of the results by adjusted according to GOLD classification, age, forced expiratory volume in 1 s (FEV_1_), smoking status, body mass index, inhaled corticosteroids/long-acting β_2_ agonist (ICS/LABA).

Factors with *p* < 0.2 in the univariate analysis were included in multivariate logistic regression analysis to determine the independent predictors of osteoporosis. Statistical significance was set at *p* < 0.05. All statistical analyses were performed using the Stata/MP 16.0 Mac.

## 3. Results

**Figure 1:**
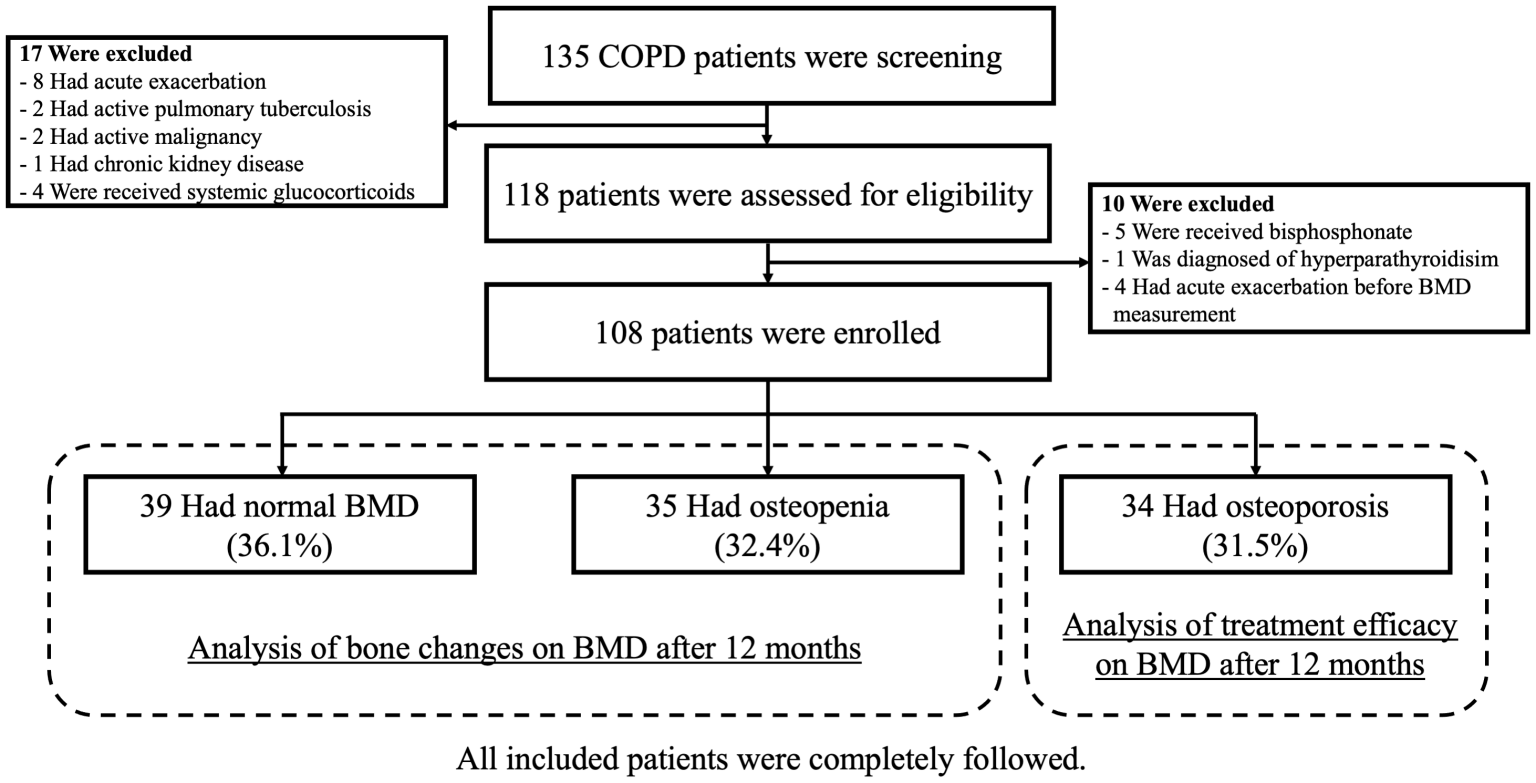
Patient enrollment and follow-up diagram.

Altogether, 118 patients with stable COPD were screened, and 108 patients were included. After BMD measurement was performed, 34 (31.5%), 35 (32.4%), and 39 (36.1%) patients were categorized into the osteoporosis, osteopenia, and normal groups, respectively (Figure 1). The patients in the osteoporosis group received pharmacotherapy. All patients were followed up.

### 3.1. Prevalence of osteoporosis

The overall prevalence of osteoporosis and osteopenia according to the *T*-score at either L2–4 or both femoral necks were 31.5 and 32.4%, respectively.

### 3.2. Patient characteristics

The baseline characteristics are presented in Table 1. The median (IQR) age of the overall population was 72 (66.0, 78.5) years. The median age of the osteoporosis and osteopenia groups was similar but significantly older than that of the normal BMD group (76.0 (72.0, 80.0) vs. 76 (65.0, 82.0) vs. 69 (64.0, 72.0) years, *p* = 0.001, respectively). Most of the patients were men (97.4%). Interestingly, the median weight, height, and BMI were significantly lower in the osteoporosis group. Sixteen COPD patients (45.7%) with osteopenia also had diabetes mellitus, which was higher than in the other groups (*p* < 0.001).

**Table 1:** Demographic and baseline patient characteristics.

Patient’s characteristics	Normal (*n* = 39)	Osteopenia (*n* = 35)	Osteoporosis (*n* = 34)	Total (*n* = 108)	value of *p*
Age (year), median (IQR)	69.0 (64.0, 72.0)	76.0 (65.0, 82.0)	76.0 (72.0, 80.0)	72.0 (66.0, 78.5)	0.001
Male, *n* (%)	38 (97.4)	32 (91.4)	30 (88.2)	100 (92.6)	0.310
Weight (kg), median (IQR)	68.0 (57.0, 74.0)	58.0 (52.0, 66.0)	47.9 (45.0, 55.0)	57.2 (48.9, 68.5)	<0.001
Height (cm), mean ± SD	163.0 ± 5.2	164.2 ± 6.4	159.3 ± 6.4	162.2 ± 6.3	0.001
BMI (kg/m^2^), median (IQR)	24.8 (21.4, 27.4)	21.8 (19.0, 23.8)	19.6 (18.3, 21.3)	21.6 (19.2, 24.8)	<0.001
BMI, Categories					
<18.5, *n* (%)	1 (2.6)	5 (14.3)	9 (26.5)	15 (13.9)	0.020
18.5–23, *n* (%)	16 (41.0)	18 (51.4)	18 (52.9)	52 (48.1)	
23–25, *n* (%)	12 (30.8)	5 (14.3)	4 (11.8)	21 (19.4)	
≥25, *n* (%)	10 (25.6)	7 (20.0)	3 (8.8)	20 (18.5)	
FEV_1_ (%), median (IQR)	71.0 (46.0, 82.0)	60.5 (47.0, 69.0)	49.5 (38.0, 61.0)	57.0 (46.0, 75.0)	0.106
FVC (%), median (IQR)	99.0 (78.0, 111.0)	86.5 (73.0, 98.0)	87.5 (74.0, 102.0)	90.0 (74.0, 103.0)	0.190
FEV_1_/FVC, mean ± SD	54.3 ± 12.3	52.2 ± 10.6	47.7 ± 12.1	51.5 ± 11.9	0.077
GOLD (FEV_1_classification), *n* (%)					
>80	14 (35.9)	4 (11.8)	6 (17.6)	24 (22.4)	0.012
50–80	13 (33.3)	23 (67.6)	11 (32.4)	47 (43.9)	
30–50	11 (28.2)	6 (17.6)	15 (44.1)	32 (29.9)	
<30	1 (2.6)	1 (2.9)	2 (5.9)	4 (3.7)	
GOLD group, *n* (%)					
A	15 (38.5)	12 (35.3)	2 (5.9)	29 (27.1)	0.001
B	14 (35.9)	16 (47.1)	22 (64.7)	52 (48.6)	
C	5 (12.8)	4 (11.8)	0 (0.0)	9 (8.4)	
D	5 (12.8)	2 (5.9)	10 (29.4)	17 (15.9)	
Smoking status					
current smoker, *n* (%)	38 (97.4)	30 (85.7)	28 (82.4)	96 (88.9)	0.095
ex-smoker, *n* (%)	1 (2.6)	5 (14.3)	6 (17.6)	12 (11.1)	
Underlying disease					
Dyslipidemia, *n* (%)	15 (38.5)	10 (28.6)	9 (26.5)	34 (31.5)	0.493
Hypertension, *n* (%)	18 (46.2)	14 (40.0)	10 (29.4)	42 (38.9)	0.338
Coronary disease, *n* (%)	4 (10.3)	5 (14.3)	6 (17.6)	15 (13.9)	0.658
Diabetes mellitus, *n* (%)	7 (17.9)	16 (45.7)	2 (5.9)	25 (23.1)	<0.001
Dose of steroid*(mg), mean ± SD	35.5	37.52	52.17	42.5	0.067
ICS used, *n* (%)	36 (92.3)	33 (94.3)	34 (100.0)	103 (95.4)	0.276
ICS dose (mg/day)					
Fluticasone 500, *n* (%)	24 (66.7)	21 (60.0)	27 (79.4)	72 (68.6)	0.196
Fluticasone 750, *n* (%)	0 (0.0)	0 (0.0)	1 (2.9)	1 (1.0)	
Budesonide 640, *n* (%)	12 (33.3)	14 (40.0)	6 (17.6)	32 (30.5)	
CAT score, mean ± SD	17.1 ± 8.7	17.2 ± 8.4	18.4 ± 9.9	17.6 ± 8.3	0.158
mMRC dyspnea score					
1, *n* (%)	26 (66.7)	17 (48.6)	2 (5.9)	45 (41.7)	<0.001
2, *n* (%)	13 (33.3)	16 (45.7)	31 (91.2)	60 (55.6)	
3, *n* (%)	0 (0.0)	2 (5.7)	0 (0.0)	2 (1.9)	
4, *n* (%)	0 (0.0)	0 (0.0)	1 (2.9)	1 (0.9)	
History of exacerbation in a previous year, *n* (%)	8 (20.5)	10 (28.5)	19 (55.8)	37 (34.3)	0.041
CRP (mg/L), mean ± SD	0.92 ± 1.33	1.62 ± 1.12	4.78 ± 1.97	2.54 ± 2.96	0.047

* dose of steroid: dose of systemic corticosteroid in the previous year.

BMI, body mass index; CAT, COPD assessment test; CRP, C-reactive protein; FEV_1_, forced expiratory volume in 1 s; FVC, forced vital capacity; GOLD, Global initiative for chronic obstructive lung disease; ICS, inhaled corticosteroid; IQR, interquartile range; mMRC, modified medical research council; SD, standard deviation; LABA, long-acting β_2_ agonist; LAMA, long-acting muscarinic antagonist; mMRC, modified medical research council.

In the osteoporosis group, 44.1% of the patients were classified as having a severe airflow limitation based on FEV_1_, and 64.7% of the patients were categorized as group B based on the GOLD assessment. Most COPD patients with osteoporosis in our study (94.1%) had a modified Medical Research Council (mMRC) ≥2, and 55% of them had a history of exacerbation in the previous year, which was significantly higher than that in the osteopenia and normal groups (28.5 and 20.2%, respectively; *p* = 0.041).

The mean CRP level was significantly higher in the osteoporosis group, 4.78 ± 1.97 mg/L than in the osteopenia group, 1.62 ± 1.12 mg/L and the normal group, 0.92 ± 1.33 mg/L (*p* = 0.047). There were no other differences in the baseline characteristics.

### 3.3. Potential predictors of osteoporosis in COPD patients

From the univariate and multivariate analyses of all baseline characteristics, age > 60 years, BMI <18 kg/m^2^, history of exacerbation in a previous year, and a CPR level > 0.6 mg/L were the significant potential predictors for osteoporosis, as shown in Table 2.

**Table 2:** Multivariate analysis of potential predictors for osteoporosis in COPD patients.

Variables	Odd ratio	95% CI	value of *p*
Age > 60 years	1.58	1.09–2.28	0.041
Low BMI (< 18 kg/m^2^)	1.64	1.36–3.01	0.039
History of exacerbation in a previous year	1.92	1.25–4.79	0.036
High CRP level (> 0.6 mg/L)	1.52	1.12–2.34	0.048

BMI, body mass index; CRP, C-reactive protein.

### 3.4. Association between BMD and GOLD groups

**Figure 2:**
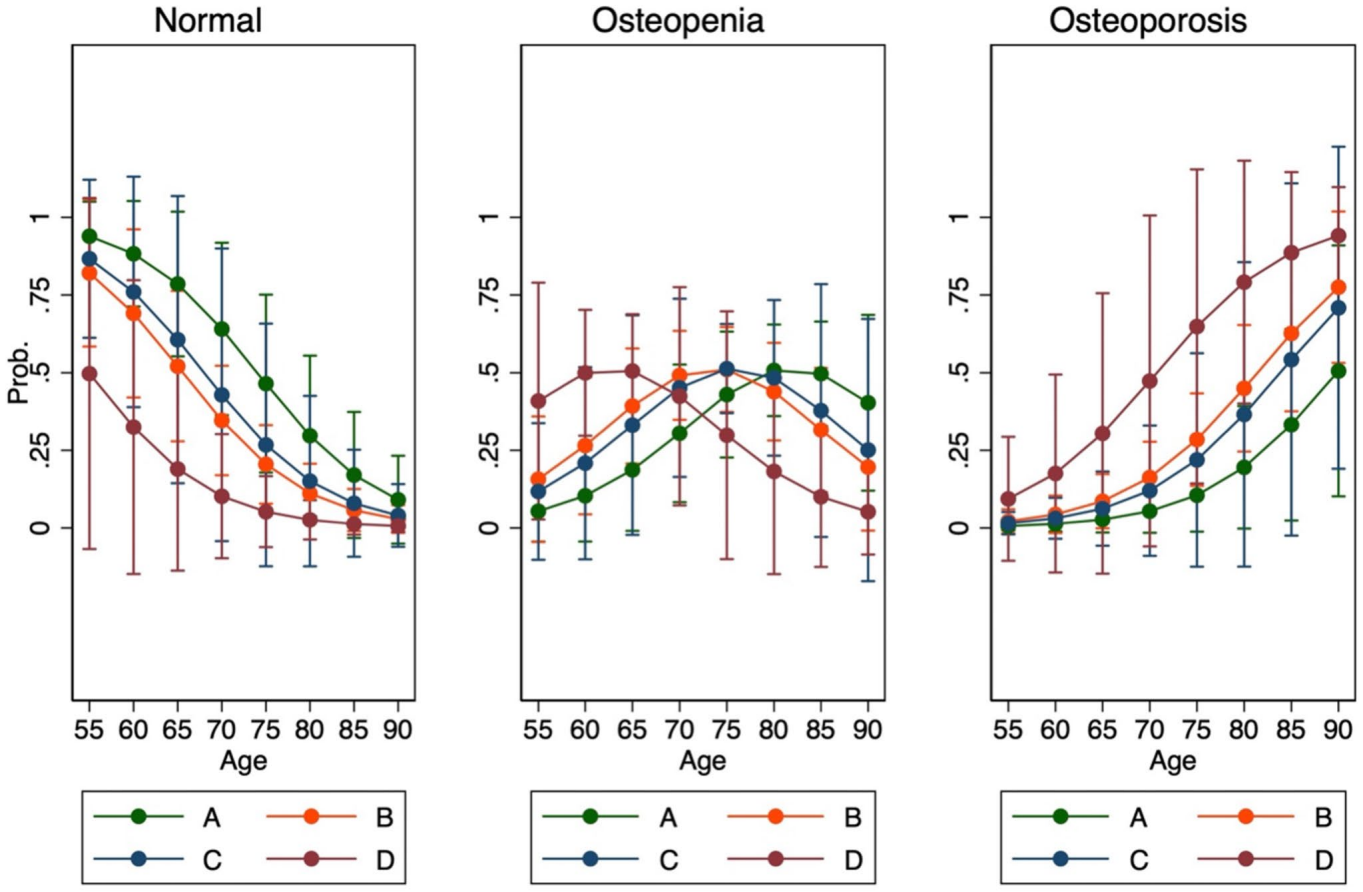
Association between BMD and GOLD groups according to age.

COPD patients in each group were classified into four GOLD groups, and the probabilities of osteoporosis and osteopenia were assessed according to age. Patients with COPD GOLD D had the highest probability of having osteoporosis, and a greater effect was seen in older age (Figure 2).

### 3.5. Dynamic changes in BMD and treatment efficacy of bisphosphonate

We examined the mean percentage change in BMD at 12 months in each group. In the normal and osteopenia groups, BMD at the lumbar spine and right and left femoral neck further declined (BMD values in the normal group were − 2.8, −4.3%, and − 3.2% and those in the osteopenia group were − 1.8, −5.1%, and − 3.8%, respectively). During the 12-month period, two COPD with osteopenia patients had declined BMD values at the vertebral body and turned to osteoporosis.

**Figure 3:**
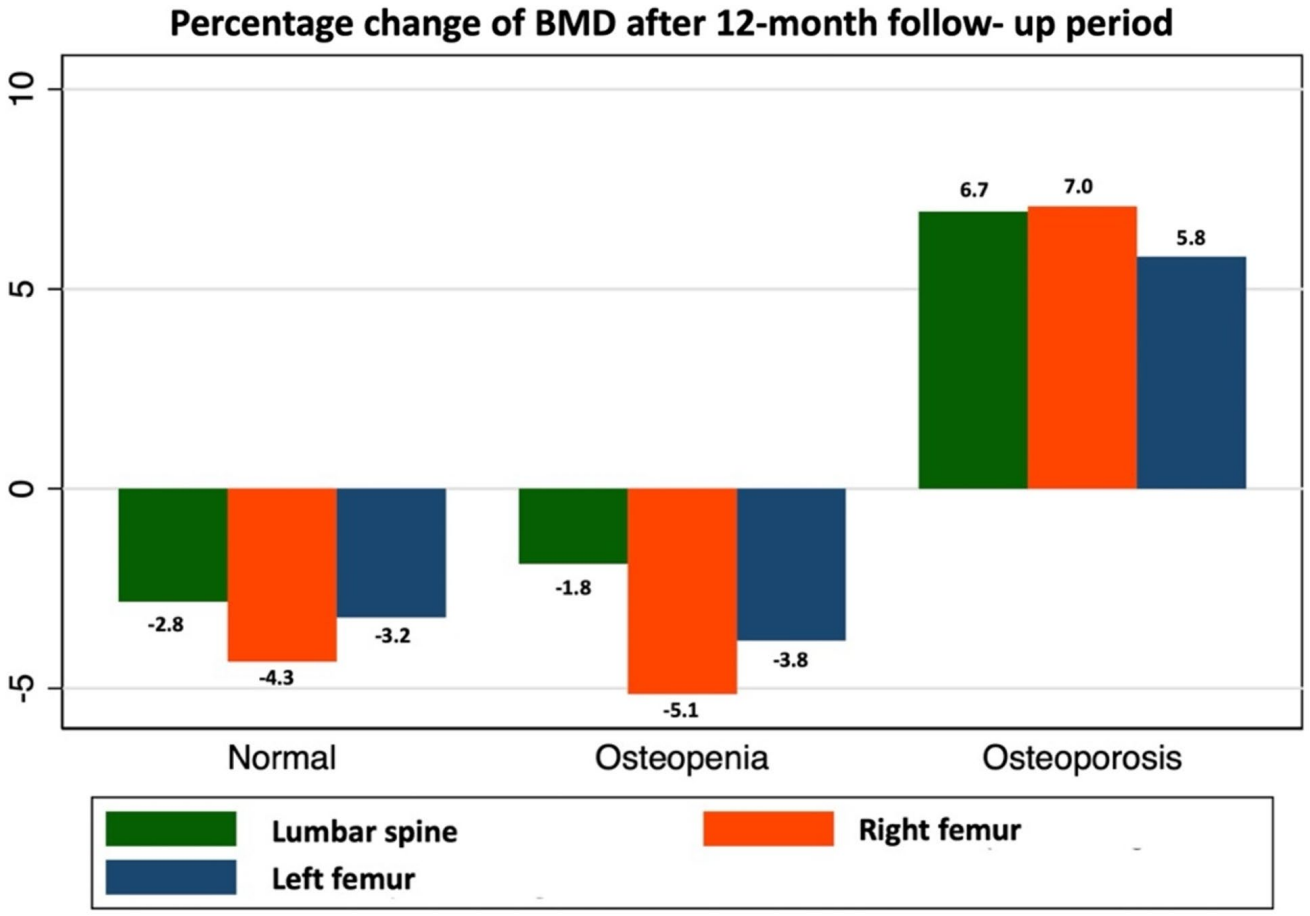
Dynamic changes in BMD in COPD patients.

Conversely, the osteoporosis group that received alendronate 70 mg/week (with calcium and vitamin D) showed a statistically significant improvement in BMD of 6.7, 7.0, and 5.8% in the lumbar spine, right femur, and left femur, respectively (*p* < 0.001), as shown in Figure 3.

Over the 12-month study period, 12 patients (35.3%) in the osteoporosis group had new vertebral and femoral fractures, whereas no fracture was reported in the non-osteoporosis group (*p* < 0.001).

## 4. Discussion

This study has four important findings. First, the prevalence of osteoporosis in COPD was 31.5% and that of osteopenia was 32.5%. The second is that the potential risk factors for osteoporosis were age > 60 years, BMI <18 kg/m^2^, history of exacerbation in the previous year, and a CRP level > 0.6 mg/L. Third, COPD GOLD D patients with older age had the highest probability of osteoporosis. Lastly, osteoporotic COPD patients who received anti-resorptive treatment had a statistically significant improvement in BMD after prospective follow-up.

To the best of our knowledge, osteoporosis is an important comorbidity of COPD that can lead to poor health status. This study found that one-third of Thai COPD patients had osteoporosis and nearly two-thirds had low BMD. The prevalence in our study was similar to that in other studies, which was reported as 31–40%, (8–10, 12) and also corresponded with a prior study in Thai COPD patients by Rittayamai et al. (11) However, our study also included women, which can cause a higher prevalence due to hormonal effects. The prevalence of the Thai male population was 12.6% as reported by Pongchiyakul et al. (22) However, we found a prevalence greater by 2.5 times; this difference might be attributed to the younger population in their study (mean age of 51 ± 16 years). The effect of aging on bone metabolism could result in a high prevalence of osteoporosis in the elderly, and most patients were diagnosed at an older age.

Advanced age was also reported as an associated factor for osteoporosis in COPD in a previous study (23). Low BMI has also been documented as a risk factor for osteoporosis in numerous studies, (8, 10, 11, 24, 25) which could be explained by various mechanisms, including hormonal levels, changes in body composition, and mechanical loading on the weight-bearing bones that facilitate bone formation and are also correlated with systemic inflammation in COPD (7, 26, 27). History of exacerbation in the previous year increased the risk of osteoporosis in our patients. Although this factor remains controversial in most studies, a few trials have reported that COPD exacerbations are independently associated with osteoporosis progression (28, 29). COPD exacerbation is hypothesized to be a risk factor for osteoporosis because of important factors such as systemic inflammation, systemic corticosteroid prescription, and physical inactivity during acute exacerbation (30, 31). Systemic inflammation occurs at all stages of COPD, especially during acute exacerbations. To our knowledge, systemic corticosteroid usage is a common cause of osteoporosis and fracture according to a meta-analysis of 42,000 subjects (32). The inflammatory process is demonstrated by increased production of various cytokines, including the bone resorption marker collagen type I β-isomerized C-terminal telopeptide (beta CL), CRP, matrix metalloproteinase-9 (MMP-9), and tissue inhibitor of metalloproteinase-1 (TIMP-1). During exacerbation, the total oxidative status (TOS) was higher than that in the stable state (*p* < 0.05) (33). Unsurprisingly, we found that the CRP level, an inflammatory reactive cytokine, was higher in patients with osteoporosis than in those without osteoporosis. This factor is similar to that in another study in Thailand (11). Therefore, our results support the hypothesis that systemic inflammation is responsible for the high prevalence of osteoporosis in COPD patients.

Systemic and inhaled corticosteroid usage were not claimed as potential predictors of osteoporosis in this study, which is consistent with the TORCH study by Ferguson et al. (9) and the recent systematic and meta-analysis by Chen et al. (8) Severe COPD patients had a higher risk of osteoporosis about four times, as well as the lower FEV_1_ and FEV_1_/FVC ratio tended to have a higher risk of getting osteoporosis from a longitudinal study by Bitar et al. (13). However, the severity of airflow limitation could not be found as a significant risk factor for osteoporosis in our study, which was different from that reported in previous studies, (10, 12) but the same as that reported in two recent systematic and meta-analysis studies (8, 24).

Additionally, a more rapid decline in BMD was observed in the lumbar, right, and left femur in our COPD patients than in the non-COPD osteoporotic patients without treatment (34). Most patients in this study were males. Men have a slower bone mineral density loss than post-menopausal women because of hormonal effect. From the previous report, the loss of bone mineral density in men will occur after the sixth decade of life about 0.5–1% per year (35).

We also assessed the treatment efficacy of bisphosphonate; alendronate has a beneficial effect on BMD, including that in the lumbar spine and both femurs, which was similar to that reported in a prospective observational study from Japan (36). An earlier RCT by Smith et al. (18) evaluated the effect of bisphosphonate in patients with airway disease (mostly asthma patients), and the results demonstrated a significant benefit on BMD only at the lumbar spine. These results could reflect the benefit of alendronate in preventing rapid bone decline in COPD as well as in postmenopausal, male, and glucocorticoid-induced osteoporosis. From our study, in COPD patients with normal BMD and osteopenia group also showed a significant decline after only 1 year period. These findings could be implied to general practice such as routine BMD screening and close follow-up, early initiation of vitamin D and calcium supplements, early considering anti-resorptive drugs if possible.

To our knowledge, this is the first prospective cohort study to demonstrate many aspects of osteoporosis in Thai COPD patients. In this study, we minimized confounding factors as much as we could by using seemingly unrelated regression (SUR) analysis. The confounders including GOLD classification, age, FEV_1_, smoking status, body mass index, ICS/LABA used were already adjusted. However, gender was not adjusted due to the small number of females in our study. These results could alert pulmonologists to the importance of osteoporosis. Routine BMD screening can lead to early diagnosis and initiation of treatment. This issue will prevent fractures and improve the quality of life and prognosis. However, our study has some limitations. First, our study was not population-based with age-and sex-matched controls. Second, a 1-year-follow up period might not be sufficient to represent the BMD change, but it can display a trend of changes, as well as this period was not long enough to confirm the long-term benefit of bisphosphonate and observe its side effects. Therefore, further studies should be conducted on population-based age-sex-matched control patients and extend the duration of treatment to confirm long-term benefits and side effects.

## 5. Conclusion

The overall prevalence of osteoporosis is high in Thai COPD patients. Advanced age, lower BMI, history of exacerbation, and high CRP levels were potential predictors. A rapid decline in BMD was observed in COPD patients without treatment, whereas alendronate prevented further BMD decline in osteoporotic COPD patients.

## Data availability statement

The raw data supporting the conclusions of this article will be made available by the authors, without undue reservation.

## Ethics statement

The studies involving humans were approved by the Office of Human Research Ethics Committee at the Faculty of Medicine, Prince of Songkla University, Thailand (REC.57-225-14-1). All patients provided written informed consent to participate in this study prior to enrollment.

## Author contributions

PK, WK, and SG: conceptualisation, methodology, and data interpretation. NC and PS: data collection and investigation. PK and SG: data analysis. PK and WK: validation. PK: data curation and writing-original draft preparation. WK: writing – review and editing, supervision, and project administration. All authors contributed to the article and approved the final version of the manuscript.

## Funding

This work was supported by the Faculty of Medicine, Prince of Songkla University (REC.57-225-14-1).

## Conflict of interest

The authors declare that the research was conducted in the absence of any commercial or financial relationships that could be construed as a potential conflict of interest.

## Publisher’s note

All claims expressed in this article are solely those of the authors and do not necessarily represent those of their affiliated organizations, or those of the publisher, the editors and the reviewers. Any product that may be evaluated in this article, or claim that may be made by its manufacturer, is not guaranteed or endorsed by the publisher.

## References

[1] The top 10 causes of death. Available at: https://www.who.int/news-room/fact-sheets/detail/the-top-10-causes-of-death (Accessed January 22, 2022).

[2] Global initiative for chronic obstructive lung disease. Global strategy for the diagnosis, management, and prevention of chronic obstructive pulmonary disease (2021). Available at: https://goldcopd.org/ (Accessed January 22, 2022).

[3] Compston JE McClung MR Leslie WD. Osteoporosis. Lancet. (2019) 393:364–76. doi: 10.1016/S0140-6736(18)32112-3 30696576

[4] Inoue D Watanabe R Okazaki R. COPD and osteoporosis: links, risks, and treatment challenges. Int J Chron Obstruct Pulmon Dis. (2016) 11:637–48. doi: 10.2147/COPD.S79638 27099481 PMC4820217

[5] de Sire A Lippi L Aprile V Calafiore D Folli A D’Abrosca F. Pharmacological, nutritional, and rehabilitative interventions to improve the complex Management of Osteoporosis in patients with chronic obstructive pulmonary disease. A Narrative Review J Pers Med. (2022) 12:1–23. doi: 10.3390/jpm12101626 PMC9604650 36294765

[6] Lippi L Folli A Curci C D’Abrosca F Moalli S Mezian K. Osteosarcopenia in patients with chronic obstructive pulmonary diseases: which pathophysiologic implications for rehabilitation? Int J Environ Res Public Health. (2022) 19:1–18. doi: 10.3390/ijerph192114314, PMID: 36361194 PMC9657186

[7] Sarkar M Bhardwaj R Madabhavi I Khatana J. Osteoporosis in chronic obstructive pulmonary disease. Clin Med Insights Circ Respir Pulm Med. (2015) 9:CCRPM.S22803–21. doi: 10.4137/CCRPM.S22803, PMID: 25788838 PMC4358421

[8] Chen YW Ramsook AH Coxson HO Bon J Reid WD. Prevalence and risk factors for osteoporosis in individuals with COPD: a systematic review and meta-analysis. Chest. (2019) 156:1092–110. doi: 10.1016/j.chest.2019.06.036 31352034

[9] Ferguson GT Calverley PMA Anderson JA Jenkins CR Jones PW Willits LR. Prevalence and progression of osteoporosis in patients with COPD: results from the towards a revolution in COPD health study. Chest. (2009) 136:1456–65. doi: 10.1378/chest.08-3016 19581353

[10] Graat-Verboom L Wouters EFM Smeenk FWJM Van Den BBEEM Lunde R Spruit MA. Current status of research on osteoporosis in COPD: a systematic review. Eur Respir J. (2009) 34:209–18. doi: 10.1183/09031936.50130408, PMID: 19567604

[11] Rittayamai N Chuaychoo B Sriwijitkamol A. Prevalence of osteoporosis and osteopenia in Thai COPD patients. J Med Assoc Thail Chotmaihet Thangphaet. (2012) 95:1021–7. 23061305

[12] Abbasi M Zohal M Atapour B Yazdi Z. Prevalence of osteoporosis and its risk factors in men with COPD in Qazvin. Int J Chronic Dis. (2016) 2016:1–6. doi: 10.1155/2016/4038530, PMID: 27774508 PMC5059585

[13] Bitar AN Sulaiman SAS Ali IABH Khan AH. Prevalence, risk assessment, and predictors of osteoporosis among chronic obstructive pulmonary disease patients. J Adv Pharm Technol Res. 2021/10/20 ed. (2021) 12:395–401. doi: 10.4103/japtr.japtr_98_21, PMID: 34820316 PMC8588927

[14] Graumam RQ Pinheiro MM Nery LE Castro CHM. Increased rate of osteoporosis, low lean mass, and fragility fractures in COPD patients: association with disease severity. Osteoporos Int. (2018) 29:1457–68. doi: 10.1007/s00198-018-4483-z, PMID: 29564475

[15] Camacho PM Petak SM Binkley N Diab DL Eldeiry LS Farooki A. American Association of Clinical Endocrinologists/American College of Endocrinology Clinical Practice Guidelines for the diagnosis and treatment of postmenopausal osteoporosis—2020 update. Endocr Pract. (2020) 26:1–46. doi: 10.4158/GL-2020-0524SUPPL, PMID: 32427503

[16] Reid IR. A broader strategy for osteoporosis interventions. Nat Rev Endocrinol. (2020) 16:333–9. doi: 10.1038/s41574-020-0339-7, PMID: 32203407

[17] Reid IR. Revisiting osteoporosis guidelines. Lancet Diabetes Endocrinol. (2021) 9:805–6. doi: 10.1016/S2213-8587(21)00283-7, PMID: 34688355

[18] Smith BJ Laslett LL Pile KD Phillips PJ Phillipov G Evans SM. Randomized controlled trial of alendronate in airways disease and low bone mineral density. Chron Respir Dis. (2004) 1:131–7. doi: 10.1191/1479972304cd025oa, PMID: 16281654

[19] Global Initiative for Chronic Obstructive Lung Disease (GOLD). (2017). Global strategy for the diagnosis, management and prevention of chronic obstructive pulmonary disease [Internet]. Available at: https://goldcopd.org/ (Accessed January 22, 2022).

[20] Prevention and management of osteoporosis. World Health Organ Tech Rep Ser. (2003) 921:1–164. PMID: 15293701

[21] Ngamjarus C Chongsuvivatwong V McNeil E. n4Studies: sample size calculation for an epidemiological study on a smart device. Siriraj Med J. (2016)

[22] Pongchaiyakul C Apinyanurag C Soontrapa S Soontrapa S Pongchaiyakul C Nguyen TV. Prevalence of osteoporosis in Thai men. J Med Assoc Thail Chotmaihet Thangphaet. (2006) 89:160–9. 16579001

[23] Lee SH Kwon HY. Prevalence of osteoporosis in Korean patients with chronic obstructive pulmonary disease and their health-related quality of life according to the Korea National Health and nutrition examination survey 2008–2011. J Bone Metab. (2017) 24:241–8. doi: 10.11005/jbm.2017.24.4.241, PMID: 29259964 PMC5734950

[24] Bitar AN Syed Sulaiman SA Ali IAH Khan I Khan AH. Osteoporosis among patients with chronic obstructive pulmonary disease: systematic review and Meta-analysis of prevalence, severity, and therapeutic outcomes. J Pharm Bioallied Sci. (2019) 11:310–20. doi: 10.4103/jpbs.JPBS_126_19 31619912 PMC6791086

[25] Lloyd JT Alley DE Hawkes WG Hochberg MC Waldstein SR Orwig DL. Body mass index is positively associated with bone mineral density in US older adults. Arch Osteoporos. (2014) 9:175. doi: 10.1007/s11657-014-0175-2, PMID: 24664472

[26] Rosen CJ Klibanski A. Bone, fat, and body composition: evolving concepts in the pathogenesis of osteoporosis. Am J Med. (2009) 122:409–14. doi: 10.1016/j.amjmed.2008.11.027, PMID: 19375545

[27] Montalcini T Romeo S Ferro Y Migliaccio V Gazzaruso C Pujia A. Osteoporosis in chronic inflammatory disease: the role of malnutrition. Endocrine. (2013) 43:59–64. doi: 10.1007/s12020-012-9813-x, PMID: 23055015

[28] Hattiholi J Gaude GS. Prevalence and correlates of osteoporosis in chronic obstructive pulmonary disease patients in India. Lung India Off Organ Indian Chest Soc. (2014) 31:221–7. doi: 10.4103/0970-2113.135759 PMC4129592 25125807

[29] Kiyokawa H Muro S Oguma T Sato S Tanabe N Takahashi T. Impact of COPD exacerbations on osteoporosis assessed by chest CT scan. COPD. (2012) 9:235–42. doi: 10.3109/15412555.2011.650243, PMID: 22360380 PMC3399638

[30] Groenewegen KH Postma DS Hop WCJ Wielders PLML Schlösser NJJ Wouters EFM. Increased systemic inflammation is a risk factor for COPD exacerbations. Chest. (2008) 133:350–7. doi: 10.1378/chest.07-1342 18198263

[31] Wouters EFM Groenewegen KH Dentener MA Vernooy JHJ. Systemic inflammation in chronic obstructive pulmonary disease: the role of exacerbations. Proc Am Thorac Soc. (2007) 4:626–34. doi: 10.1513/pats.200706-071TH 18073394

[32] Kanis JA Johansson H Oden A Johnell O de Laet C Melton LJ III. A Meta-analysis of prior corticosteroid use and fracture risk. J Bone Miner Res. (2004) 19:893–9. doi: 10.1359/JBMR.040134, PMID: 15125788

[33] Stanojkovic I Kotur-Stevuljevic J Spasic S Milenkovic B Vujic T Stefanovic A. Relationship between bone resorption, oxidative stress and inflammation in severe COPD exacerbation. Clin Biochem. (2013) 46:1678–82. doi: 10.1016/j.clinbiochem.2013.08.003, PMID: 23954853

[34] Ho YV Frauman AG Thomson W Seeman E. Effects of alendronate on bone density in men with primary and secondary osteoporosis. Osteoporos Int. (2000) 11:98–101. doi: 10.1007/PL00004182, PMID: 10793867

[35] Melton LJ III Khosla S Achenbach SJ O’Connor MK O’Fallon WM Riggs BL. Effects of body size and skeletal site on the estimated prevalence of osteoporosis in women and men. Osteoporos Int. (2000) 11:977–83. doi: 10.1007/s001980070037, PMID: 11193251

[36] Kameyama N Chubachi S Sasaki M Tsutsumi A Irie H Sakurai K. Predictive and modifying factors of bone mineral density decline in patients with COPD. Respir Med. (2019) 148:13–23. doi: 10.1016/j.rmed.2019.01.005, PMID: 30827469

